# Translation, Cultural Adaptation, and Psychometric Validation of the Greek Version of the Standardized Cosmesis and Health Nasal Outcomes Survey (SCHNOS)

**DOI:** 10.7759/cureus.99608

**Published:** 2025-12-19

**Authors:** Argyro Kypraiou, Giorgos Sideris, Alexander Delides, Thomas Nikolopoulos, Petros V Vlastarakos

**Affiliations:** 1 Department of Functional and Reconstructive Nasal Surgery, MITERA Infirmary, Athens, GRC; 2 2nd ENT Department, Attikon University Hospital, National and Kapodistrian University of Athens, Athens, GRC

**Keywords:** cultural adaptation, greek translation, patient-reported outcome measures, rhinoplasty, schnos

## Abstract

Introduction: Reliable assessment of both functional and aesthetic outcomes is essential in rhinoplasty. The Standardized Cosmesis and Health Nasal Outcomes Survey (SCHNOS) is widely used internationally, but no validated Greek version was previously available. The objective of this prospective comparative observational study is to translate, culturally adapt, and validate the SCHNOS for Greek-speaking patients.

Methods: The SCHNOS underwent forward-backward translation, expert review, and pilot testing. A total of 49 participants were included (24 rhinoplasty patients and 25 controls). Internal consistency (Cronbach’s α), test-retest reliability (intraclass correlation coefficient (ICC)), and discriminant validity (Mann-Whitney U test) were assessed.

Results: Internal consistency was excellent (SCHNOS-O α = 0.90; SCHNOS-C α = 0.98). Test-retest reliability was also excellent (ICC = 0.990 and 0.995). Rhinoplasty patients scored significantly higher on both subscales than controls (*P* < 0.001), confirming discriminant validity.

Conclusions: The Greek SCHNOS is a reliable and valid patient-reported measure for functional and aesthetic rhinoplasty outcomes.

## Introduction

Rhinoplasty is one of the most common procedures in plastic surgery, as the nose is the most prominent feature in the face, and may attract attention in case of external deviation or malformation [1-2]. In addition, breathing and/or snoring problems exacerbated by septal deviation or inferior turbinate enlargement may also represent an important issue, frequently perplexed by the form and shape of the nose, or the quality of the overlying skin [3-4].

Hence, functional rhinoplasty has emerged as a highly popular operation in facial plastic surgery; however, there is a need for a patient-based evaluation, pertaining to both the preoperative situation and the postoperative outcome, about the functional and aesthetic domains.

The Standardized Cosmesis and Health Nasal Outcomes Survey (SCHNOS) is a validated 10-item patient-reported outcome measure (PROM) designed to assess both functional and cosmetic aspects of nasal appearance in patients undergoing rhinoplasty [5].

The present study aims to translate, culturally adapt, and psychometrically validate the Greek version of the SCHNOS by evaluating its internal consistency, test-retest reliability, and known-groups validity in Greek-speaking populations.

## Materials and methods

The questionnaire

The SCHNOS scale consists of 10 items, which attempt to describe the severity of nasal obstruction and the extent of aesthetic dissatisfaction following rhinoplasty. The SCHNOS scale items cover two domains, producing two separate scores: SCHNOS-O (obstruction, items 1-4) and SCHNOS-C (cosmesis, items 5-10). Each item is scored on a six-point Likert scale (0= no problem, 5= extreme problem). Participants completed socio-demographic questions regarding age (years) and sex (male/female).

Translation

The SCHNOS was translated and culturally adapted according to the “Minimal Translation Criteria” [6-7]. The process included the independent translation of the original English version into Greek by two bilingual native Greek physicians with experience in otolaryngology (Forward Translation). The two forward translations were subsequently compared and reviewed by a third bilingual native Greek reviewer, who resolved discrepancies and produced a mutually agreed Greek version (First Reconciliation Version). This version then underwent translation back into English by a professional bilingual native English-speaking translator who was blinded to the original questionnaire (Backward Translation), to identify potential discrepancies.

Following this process, the Greek version of the questionnaire was pilot tested in a sample of 10 patients from the target population to assess clarity, comprehension, and cultural relevance, thereby confirming its apparent validity (Face Validity) and ensuring that no items resulted in incomplete or misleading responses [8].

In addition, the questionnaire was evaluated by three experts in the field of cosmesis and nasal health to assess content validity and propose potential improvements [9-10]. Items with a Content Validity Ratio (CVR) > 0.70 were retained in the final version of the instrument, while items not meeting this threshold were appropriately revised. The Content Validity Index (CVI) for the instrument as a whole was also calculated, with values > 0.80 considered acceptable.

Setting and participants

A prospective comparative study was conducted in the ENT Department of a tertiary University Hospital. The collection of data took place between April and September 2025. A convenience sample was used. The study involved 25 patients receiving functional rhinoplasty and 25 ENT patients not suffering from rhinologic problems. The study protocol was approved by the Ethics Committee of the affiliated university (IRB 257/31-03-2025). Patients provided written informed consent before enrollment in the study.

Reliability-validity

Internal consistency was assessed by Cronbach’s alpha (α). A Cronbach α coefficient >0.7 indicates sufficient reliability for research purposes and suggests that items are interdependent and homogeneous in terms of the construct they measure. A Cronbach α >0.8 is desirable for clinical applications. Power analysis estimated that for a 10-item questionnaire with a minimum acceptable Cronbach’s α of 0.5 and an expected Cronbach’s α of 0.8, the minimum sample size would be 46 people (23 each group) to obtain results at the p= 0.05 level of statistical significance, with 80% certainty.

Intra-rater reliability was determined by calculating the intraclass correlation coefficient (ICC) on the initial assessment and the reassessment after a two-week interval, for each item separately, in a random subgroup of the participants. Values below 0.5 indicate poor reliability, between 0.5 and 0.75 indicate moderate reliability, and between 0.75 and 0.9 indicate moderate and good reliability, respectively, and any value above 0.9 shows excellent reliability. Power analysis again indicated that the minimum sample size in the intra-rater reliability analysis, for the minimum acceptable reliability of 0.8, with an expected drop out rate of 10% would be 19 people to obtain results at the *P *= 0.05 level of statistical significance, with 99% certainty; hence, 20 randomly selected people (10 from each group) were included in the respective analysis.

In the absence of a gold standard to test construct validity, a validation study can examine known group (or outgroup) validity. Following the aforementioned approach, the questionnaire can be administered to two groups that are known to have, or should reasonably have, different levels of the construct to confirm whether the hypothesized difference is reflected in the scores of the two groups. Mann-Whitney U test estimated that in order to obtain a difference between the rhinoplasty group and the non-rhinologic ENT patients at the p= 0.05 level of statistical significance, with 80% certainty, and a minimum effect size of 0.8, the minimum sample size should be at least 42 people (21 each group).

Finally, 49 patients (25 in the non-rhinologic ENT group and 24 in the rhinoplasty group) opted to participate in the study.

Data analysis

Statistical analyses were conducted using IBM SPSS (version 25, IBM Corp., Armonk, NY). Continuous variables were expressed as median and quartiles 1 and 2 (Q1, Q2). Categorical variables were expressed as numbers and percentages (*n*, %). Statistical significance was set at *P* < 0.05.

## Results

Forty-nine participants were included in the study; among them, 37 (75.5%) were females, and 12 (24.5%) were males. The respective ages ranged between 16 and 59 years (median = 41, Q1 = 31, Q2 = 49) (Table 1).

**Table 1:** Sample characteristics.

Sex, *n* (%)	Healthy asymptomatic group	Rhinoplasty group	Total	*P*-value
Female	17 (70.8)	20 (80)	37 (75.5)	0.456
Male	7 (29.2)	5 (20)	12 (24.5)	
Age Mdn (Q1, Q2)	42 (28.5, 49)	35 (32, 49)	41 (31, 49)	0.849

Regarding internal consistency, Cronbach's alpha coefficients for the SCHNOS-O and SCHNOS-C domains of the Greek version of SCHNOS were found to be 0.90 and 0.98, respectively, indicating high internal consistency. The individual item contribution to each SCHNOS subscale is reported (Table 2).

**Table 2:** Contribution of each item to the SCHNOS-O and SCHNOS-C subscales. SCHNOS, Standardized Cosmesis and Health Nasal Outcomes Survey

	Scale mean if item deleted	Scale variance if item deleted	Corrected item-total correlation	Squared multiple correlation	Cronbach's alpha if item deleted
SCHNOS-O					
SCHNOS (item1)	7.65	22.815	0.848	0.721	0.847
SCHNOS (item2)	7.78	21.344	0.845	0.728	0.845
SCHNOS (item3)	8.14	25.625	0.657	0.478	0.912
SCHNOS (item4)	7.53	22.213	0.770	0.655	0.875
SCHNOS-C					
SCHNOS (item5)	12.18	97.611	0.861	0.796	0.977
SCHNOS (item6)	11.80	94.124	0.934	0.895	0.970
SCHNOS (item7)	11.80	94.541	0.911	0.887	0.972
SCHNOS (item8)	11.59	93.622	0.913	0.883	0.972
SCHNOS (item9)	11.76	94.814	0.953	0.937	0.968
SCHNOS (item10)	11.59	92.247	0.949	0.927	0.968

The ICCs, for each item separately, between the initial assessment and the reassessment of the test, ranged between 0.949 and 0.994. The ICCs for the SCHNOS-O and SCHNOS-C subscales were 0.990 and 0.995, respectively (Table 3).

**Table 3:** Intraclass correlation coefficients for each item separately. CI, confidence interval

	Intraclass correlation coefficient	95% CI
SCHNOS (item1)	0.949	0.875-0.979
SCHNOS (item2)	0.982	0.954-0.993
SCHNOS (item3)	0.975	0.938-0.990
SCHNOS (item4)	0.974	0.936-0.990
SCHNOS (item5)	0.970	0.926-0.988
SCHNOS (item6)	0.986	0.965-0.994
SCHNOS (item7)	0.987	0.967-0.995
SCHNOS (item8)	0.994	0.985-0.998
SCHNOS (item9)	0.980	0.952-0.992
SCHNOS (item10)	0.965	0.914-0.986
SCHNOS-O (total score)	0.990	0.976-0.996
SCHNOS-C (total score)	0.995	0.989-0.998

Regarding construct validity, the Mann-Whitney U test showed that SCHNOS-O scores were significantly higher in the rhinoplasty (median = 15) compared to the non-rhinologic ENT group (median = 4.5) (*P* < 0.001). Regarding the SCHNOS-C scores, these were found again significantly higher in the rhinoplasty group (median = 25) than in their non-rhinologic ENT counterparts (median = 0) (*P* < 0.001).

## Discussion

This study aimed to translate, culturally adapt, and validate the Greek version of the SCHNOS, a widely used patient-reported outcome measure for assessing both functional and cosmetic results following rhinoplasty [1]. The Greek edition demonstrated excellent internal consistency, with Cronbach’s α values of 0.90 for SCHNOS-O and 0.98 for SCHNOS-C. These values are comparable to previously validated versions in Chinese, Polish, Japanese, Brazilian Portuguese, European Portuguese, Italian, Turkish, Spanish, Arabic, Persian, and French populations, confirming the robustness of the underlying psychometric structure across diverse cultural settings [11-21]. Notably, the Greek version exhibited among the highest reported reliability values, particularly in the cosmetic domain (SCHNOS-C), suggesting that the aesthetic dimension of nasal appearance is perceived with a high degree of internal coherence among Greek patients (Table 4).

**Table 4:** Cross-cultural adaptations and psychometric characteristics of the SCHNOS across different language versions. SCHNOS, Standardized Cosmesis and Health Nasal Outcomes Survey

Language	Author	Sample	Cronbach’s α		Comments
			SCHNOS-O	SCHNOS-C	
Chinese [11]	Chen et al.	110	0.81	0.92	Showed cultural appropriateness and strong reliability, especially for functional outcome assessment.
Polish [12]	Jadczak et al.	31	0.888	0.883	Confirmed the two-domain structure and reliable use of SCHNOS
Japanese [13]	Takeuchi et al.	357	0.96	0.93	Confirmed cultural suitability and strong reliability
Portuguese (European) [14]	Machado et al.	58	0.93	0.95	Confirmed reliable functional and aesthetic assessment
Italian [15]	Battista et al.	411	0.90	0.95	Confirmed cultural suitability and reliable use
Portuguese (Brazilian) [16]	Tunes et al.	58	0.84	0.93	Credible internal consistency and reproducibility
Turkish [17]	Gode et al.	188	0.94	9.97	Confirmed clear discrimination between rhinoplasty patients and controls, supporting cultural appropriateness and face validity
Spanish [18]	Perez-Garcia et al.	86	0.84	0.94	Demonstrated strong conceptual equivalence and measurement properties
Arabic [19]	Abdelwahab et al.	37	0.86	0.89	Culturally relevant and feasible tool, demonstrating satisfactory reliability and validity across diverse Arabic-speaking populations
Persian [20]	Rahavi-Ezabadi et al.	50	0.87	0.88	Strong internal consistency and cross-cultural applicability
French [21]	Atallah et al.	165	0.93	0.95	Confirmed cultural suitability and reliable use
Greek	Present study	49	0.9	0.98	Confirmed strong cultural adequacy and demonstrated among the highest reliability values reported, particularly in the cosmetic domain

The test-retest reliability was also excellent, with ICC values exceeding 0.99 for both subscales, in line with previous validation studies across other linguistic adaptations, including the Chinese, Japanese, Turkish, Spanish, and Arabic versions [11,13,17-19]. These findings indicate that the Greek SCHNOS provides stable and reproducible measurements of patient-perceived nasal function and appearance. Moreover, the preservation of strong psychometric performance indicates that the translation and cultural adaptation process did not alter the conceptual structure or clinical interpretability of the original SCHNOS.

Construct validity was confirmed by significantly higher SCHNOS-O and SCHNOS-C scores in the rhinoplasty group compared to the non-rhinologic ENT group (p < 0.001), aligning with prior cross-cultural studies in which SCHNOS successfully differentiated symptomatic from asymptomatic populations [12-13,19].

This study is not without limitations. The use of a convenience sample from a single tertiary center and the relatively small sample size may limit the generalizability of the findings. In addition, cultural perceptions of nasal aesthetics and language-specific nuances may influence patient responses despite rigorous translation procedures. The study did not assess postoperative responsiveness or determine the minimally clinically important difference (MCID), both of which are important for interpreting longitudinal score changes in clinical practice. Furthermore, unlike the work of Kandathil et al., who documented the postoperative evolution of SCHNOS scores across predefined follow-up intervals in a large cohort, the present study did not include precise documentation of the time elapsed between surgery and questionnaire administration [22].

## Conclusions

The Greek version of SCHNOS demonstrated excellent internal consistency, reliability, and discriminant validity, supporting its use as a standardized outcome measure for assessing both functional and aesthetic aspects of rhinoplasty. It can therefore be recommended for pre- and postoperative evaluation in clinical practice. Future research should focus on determining the scale’s responsiveness and establishing MCID thresholds to further facilitate its use in surgical decision-making and training evaluation.
